# Supplementary material for: Large-Scale Purification and Characterization of Recombinant Receptor-Binding Domain (RBD) of SARS-CoV-2 Spike Protein Expressed in Yeast
Source: Vaccines (Basel). 2023 Oct 16;11(10):1602. doi: 10.3390/vaccines11101602 (PMC10610970; doi:10.3390/vaccines11101602)
Supplement: Supplementary file 1 [file vaccines-11-01602-s001.zip › vaccines-2552574-supplementary.pdf]

Supplementary Data

Large-Scale Purification and Characterization of Recombinant Receptor-Binding Domain (RBD) of SARS-CoV-2 Spike Protein Expressed in Yeast

Gaurav Nagar [1], Siddharth Jain [1], Meghraj Rajurkar [1], Rakesh Lothe [1], Harish Rao [1], Sourav Majumdar [1],
Manish Gautam [1], Sergio A. Rodriguez-Aponte [2], Laura E. Crowell [3], J. Christopher Love [3], Prajakta Dandekar [4], Amita Puranik [5], Sunil Gairola [1], Umesh Shaligram [1,*] and Ratnesh Jain [5,*]

[1] Serum Institute of India Pvt. Ltd. Hadapsar, Pune 411028, India; gaurav.nagar@seruminstitute.com (G.N.); siddharth.jain@seruminstitute.com (S.J.); meghraj.rajurkar@seruminstitute.com (M.R.); rakesh.lothe@seruminstitute.com (R.L.); harish.rao@seruminstitute.com (H.R.); sourav.majumdar@seruminstitute.com (S.M.); m.gautam@seruminstitute.com (M.G.); sunil.gairola@seruminstitute.com (S.G.)
[2] Koch Institute for Integrative Cancer Research, Massachusetts Institute of Technology, Department of Biological Engineering, Cambridge, MA, 02139, USA; sergrodz@mit.edu
[3] Koch Institute for Integrative Cancer Research, Massachusetts Institute of Technology, Department of Chemical Engineering, Cambridge, MA, 02139, USA; laura@sunflowertx.com (L.E.C.); clove@mit.edu (J.C.L.)
[4] Department of Pharmaceutical Sciences and Technology, Institute of Chemical Technology, Matunga, Mumbai 400019, India; pd.jain@ictmumbai.edu.in
[5] Department of Biological Sciences and Biotechnology, Institute of Chemical Technology, Matunga, Mumbai 400019, India; amita.puranik@nano-medicine.co.in
* Correspondence: umesh.shaligram@seruminstitute.com (U.S.); rd.jain@ictmumbai.edu.in (R.J.); Tel.: +91-20-26602510 (U.S.); +91-22-33612029 (R.J.)

Supplementary Table S1: Fermentation media

Sr. No.	Name of the component	Quantity (g/L)
1	Yeast Extract	10
2	Peptone	20
3	YNB	13.4
4	1 M Phosphate buffer pH 6	100ml/L
5	Glycerol	10 ml/L
6	Biotin 0.02%	4 ml/L

Supplementary Table S2: Fermentation dosing solutions

Sr. No.	Name of the component	Remark
1	50 % ammonia solution (alkali)	for maintaining pH
2	10% Ortho Phosphoric Acid solution (acid)	for maintaining pH
3	10% antifoam solution	to prevent the formation of foam
4	50% glycerol solution as substrate feed	as substrate feed
5	50% sorbitol as induction feed	as induction feed

Supplementary Table S3: Fermentation parameters

Sr. No.	Parameter	Setpoint	Mode
	Shake Flask -1 parameter (100 mL)		
1	Temperature	25 °C	Controlled
2	Agitation (RPM)	300	Controlled
3	Harvest OD at 600 nm	5 to 15	Process output
	Shake Flask -2 parameter (4 L)		
1	Temperature	25 °C	Controlled
2	Agitation (RPM)	300	Controlled
3	Harvest OD at 600 nm	5 to 15	Process output
	Seed fermentation parameter (170 L)		
1	Temperature	25 °C	Controlled
2	pH	6.5 ± 0.1	Controlled
3	Aeration (SLPM)	75 -120	Cascade mode with set point of DO 25% in controlled mode
4	OHP (bar)	0.2 – 1.0	
5	Agitation (RPM)	100 - 600	
6	Harvest OD at 600 nm	25 to 30	Process output
	Production fermentation parameter (1200 L)		
1	Temperature	25 °C	Controlled

2	pH	6.5 ± 0.1	Controlled
3	Aeration (SLPM)	450 to 550	Cascade mode with set point of DO 25% in controlled mode
4	OHP (bar)	0.2 to 1	
5	Agitation (RPM)	56 to 210	
6	Harvest OD at 600 nm	≥40 AU	Process output

Supplementary Table S4: LC-MS-based peptide mapping

Sample Batch No.	Retention Time (minutes)	Precursor m/z	Precursor Charge	Precursor Mass (Da)	Base Peptide Sequence	Start Residue Number	Stop Residue Number	Missed Cleavages	Theoretical Mass (Da)	Precursor Mass Error (Da)	Precursor Mass Error (ppm)	Enzyme
12681T002	30.89	557.280	2	1112.545	FASVYAWNR	16	24	0	1112.540	0.005	4.398	Trypsin
12681T002	27.24	621.326	2	1240.637	FASVYAWNRK	16	25	1	1240.635	0.002	1.640	Trypsin
12681T002	37.64	699.364	2	1396.714	FASVYAWNRKR	16	26	2	1396.736	-0.022	-15.845	Trypsin
12681T002	18.29	1329.142	2	2656.269	KRISNCVADYSVLYNSASFSTFK	25	47	2	2656.296	-0.026	-9.968	Trypsin
12681T002	40.24	1265.108	2	2528.202	RISNCVADYSVLYNSASFSTFK	26	47	1	2528.201	0.002	0.653	Trypsin
12681T002	44.85	1187.054	2	2372.093	ISNCVADYSVLYNSASFSTFK	27	47	0	2372.099	-0.006	-2.577	Trypsin
12681T002	16.71	456.219	2	910.423	CYGVSPTK	48	55	0	910.422	0.002	1.688	Trypsin
12681T002	49.48	1024.001	2	2045.988	LNDLCFTNVYADSFVIR	56	72	0	2045.988	0.000	0.042	Trypsin
12681T002	44.15	1302.128	2	2602.241	LNDLCFTNVYADSFVIRGDEVR	56	77	1	2602.249	-0.007	-2.754	Trypsin
12681T002	5.56	288.143	2	574.272	GDEVR	73	77	0	574.271	0.001	1.085	Trypsin
12681T002	13.03	450.252	2	898.490	QIAPGQTGK	78	86	0	898.487	0.003	2.788	Trypsin
12681T002	18.39	443.720	2	885.425	IADYNYK	87	93	0	885.423	0.002	2.557	Trypsin
12681T002	43.68	1567.229	2	3132.444	IADYNYKLPDDFTGCVIAWNSNNLDSK	87	113	1	3132.450	-0.006	-1.821	Trypsin
12681T002	41.84	1133.528	2	2265.042	LPDDFTGCVIAWNSNNLDSK	94	113	0	2265.037	0.005	2.215	Trypsin
12681T002	27.45	609.801	2	1217.588	VGGNYNYLYR	114	123	0	1217.583	0.005	4.250	Trypsin
12681T002	40.60	817.925	2	1633.836	VGGNYNYLYRLFR	114	126	1	1633.837	0.000	-0.134	Trypsin
12681T002	50.31	881.972	2	1761.930	VGGNYNYLYRLFRK	114	127	2	1761.931	-0.002	-1.052	Trypsin
12681T002	13.22	1367.709	2	2733.404	VGGNYNYLYRLFRKSNLKPFER	114	135	3	2733.450	-0.046	-16.797	Trypsin
12681T002	11.10	282.187	2	562.360	LFRK	124	127	1	562.359	0.001	1.193	Trypsin
12681T002	16.75	218.139	2	434.263	LFR	124	126	0	434.264	-0.001	-1.608	Trypsin
12681T002	16.40	559.822	2	1117.629	KSNLKPFER	127	135	1	1117.624	0.004	3.928	Trypsin
12681T002	19.44	495.774	2	989.533	SNLKPFER	128	135	0	989.529	0.003	3.262	Trypsin
12681T002	43.61	1019.069	2	2036.123	VVVLSFELLHAPATVCGPK	179	197	0	2036.113	0.010	5.008	Trypsin
12681T002	40.43	1083.111	2	2164.208	VVVLSFELLHAPATVCGPKK	179	198	1	2164.208	0.000	0.225	Trypsin
12681T002	38.61	1352.617	2	2703.219	KSTNGGDGGDGGDGGAHIVMVDAYKPTK	198	225	1	2703.256	-0.036	-13.468	Trypsin
12681T002	27.55	1288.589	2	2575.164	STNGGDGGDGGDGGAHIVMVDAYKPTK	199	225	0	2575.161	0.003	1.024	Trypsin
12681T002	24.97	1296.586	2	2591.157	STNGGDGGDGGDGGAHIVMVDAYKPTK	199	225	0	2591.156	0.001	0.575	Trypsin
12681T002	36.17	525.751	2	1049.488	ITNLCPFGE	1	9	0	1049.485	0.003	2.619	Glu-C
12681T002	22.80	698.316	2	1394.617	IYQAGSTPCNGVE	141	153	0	1394.614	0.004	2.568	Glu-C
12681T003	32.00	557.280	2	1112.545	FASVYAWNR	16	24	0	1112.540	0.005	4.189	Trypsin
12681T003	27.79	621.327	2	1240.638	FASVYAWNRK	16	25	1	1240.635	0.003	2.584	Trypsin
12681T003	18.39	1329.144	2	2656.273	KRISNCVADYSVLYNSASFSTFK	25	47	2	2656.296	-0.023	-8.626	Trypsin
12681T003	39.98	1265.108	2	2528.202	RISNCVADYSVLYNSASFSTFK	26	47	1	2528.201	0.001	0.580	Trypsin
12681T003	44.36	1187.056	2	2372.097	ISNCVADYSVLYNSASFSTFK	27	47	0	2372.099	-0.002	-0.870	Trypsin
12681T003	42.13	1633.266	2	3264.517	ISNCVADYSVLYNSASFSTFKCYGVSPTK	27	55	1	3264.511	0.006	1.953	Trypsin
12681T003	16.40	456.219	2	910.424	CYGVSPTK	48	55	0	910.422	0.002	2.469	Trypsin
12681T003	46.75	1024.002	2	2045.989	LNDLCFTNVYADSFVIR	56	72	0	2045.988	0.001	0.516	Trypsin
12681T003	43.86	1302.133	2	2602.252	LNDLCFTNVYADSFVIRGDEVR	56	77	1	2602.249	0.003	1.328	Trypsin
12681T003	17.79	728.379	2	1454.743	GDEVRQIAPGQTGK	73	86	1	1454.748	-0.005	-3.428	Trypsin
12681T003	5.63	288.143	2	574.271	GDEVR	73	77	0	574.271	0.000	0.243	Trypsin
12681T003	12.57	450.254	2	898.493	QIAPGQTGK	78	86	0	898.487	0.006	6.486	Trypsin
12681T003	49.79	883.945	2	1765.875	QIAPGQTGKIADYNYK	78	93	1	1765.900	-0.025	-13.937	Trypsin
12681T003	43.64	1567.232	2	3132.450	IADYNYKLPDDFTGCVIAWNSNNLDSK	87	113	1	3132.450	0.000	0.026	Trypsin
12681T003	18.48	443.721	2	885.427	IADYNYK	87	93	0	885.423	0.004	4.396	Trypsin
12681T003	41.64	1133.530	2	2265.046	LPDDFTGCVIAWNSNNLDSK	94	113	0	2265.037	0.009	3.880	Trypsin

12681T003	27.79	609.802	2	1217.590	VGGNYNYLYR	114	123	0	1217.583	0.007	6.003	Trypsin
12681T003	50.36	881.974	2	1761.933	VGGNYNYLYRLFRK	114	127	2	1761.931	0.002	1.095	Trypsin
12681T003	40.56	817.927	2	1633.838	VGGNYNYLYRLFR	114	126	1	1633.837	0.002	1.195	Trypsin
12681T003	12.74	1367.724	2	2733.433	VGGNYNYLYRLFRKSNLKPFER	114	135	3	2733.450	-0.018	-6.470	Trypsin
12681T003	10.83	282.188	2	562.362	LFRK	124	127	1	562.359	0.003	5.755	Trypsin
12681T003	15.93	218.140	2	434.266	LFR	124	126	0	434.264	0.002	4.688	Trypsin
12681T003	15.93	559.822	2	1117.630	KSNLKPFER	127	135	1	1117.624	0.006	4.966	Trypsin
12681T003	19.33	495.775	2	989.535	SNLKPFER	128	135	0	989.529	0.005	5.528	Trypsin
12681T003	43.27	1019.072	2	2036.129	VVVLSFELLHAPATVCGPK	179	197	0	2036.113	0.016	7.790	Trypsin
12681T003	40.17	1083.115	2	2164.215	VVVLSFELLHAPATVCGPKK	179	198	1	2164.208	0.007	3.272	Trypsin
12681T003	28.21	1288.586	2	2575.158	STNGGDGGDGGDGGAHIVMVDAYKPTK	199	225	0	2575.161	-0.003	-1.004	Trypsin
12681T003	25.08	1296.587	2	2591.159	STNGGDGGDGGDGGAHIVMVDAYKPTK	199	225	0	2591.156	0.003	1.184	Trypsin
12681T003	34.57	525.753	2	1049.491	ITNLCPFGE	1	9	0	1049.485	0.006	5.895	Glu-C
12681T003	22.67	698.315	2	1394.615	IYQAGSTPCNGVE	141	153	0	1394.614	0.001	0.738	Glu-C
12681T004	31.58	557.279	2	1112.544	FASVYAWNR	16	24	0	1112.540	0.003	3.054	Trypsin
12681T004	27.09	621.325	2	1240.635	FASVYAWNRK	16	25	1	1240.635	-0.001	-0.440	Trypsin
12681T004	38.00	699.362	2	1396.709	FASVYAWNRKR	16	26	2	1396.736	-0.027	-19.468	Trypsin
12681T004	18.38	1329.149	2	2656.283	KRISNCVADYSVLYNSASFSTFK	25	47	2	2656.296	-0.013	-4.765	Trypsin
12681T004	40.14	1265.107	2	2528.200	RISNCVADYSVLYNSASFSTFK	26	47	1	2528.201	0.000	-0.117	Trypsin
12681T004	42.26	1187.059	2	2372.103	ISNCVADYSVLYNSASFSTFK	27	47	0	2372.099	0.003	1.409	Trypsin
12681T004	41.55	1633.262	2	3264.510	ISNCVADYSVLYNSASFSTFKCYGVSPTK	27	55	1	3264.511	0.000	-0.136	Trypsin
12681T004	16.61	456.218	2	910.422	CYGVSPTK	48	55	0	910.422	0.000	0.153	Trypsin
12681T004	46.18	1024.003	2	2045.992	LNDLCFTNVYADSFVIR	56	72	0	2045.988	0.004	1.783	Trypsin
12681T004	43.49	1302.132	2	2602.250	LNDLCFTNVYADSFVIRGDEVR	56	77	1	2602.249	0.001	0.537	Trypsin
12681T004	5.80	288.143	2	574.271	GDEVR	73	77	0	574.271	0.000	0.269	Trypsin
12681T004	17.83	728.384	2	1454.754	GDEVRQIAPGQTGK	73	86	1	1454.748	0.006	4.306	Trypsin
12681T004	13.23	450.252	2	898.490	QIAPGQTGK	78	86	0	898.487	0.003	3.172	Trypsin
12681T004	49.60	883.942	2	1765.870	QIAPGQTGKIADYNYK	78	93	1	1765.900	-0.030	-16.999	Trypsin
12681T004	42.94	1567.228	2	3132.442	IADYNYKLPDDFTGCVIAWNSNNLDSK	87	113	1	3132.450	-0.008	-2.427	Trypsin
12681T004	18.48	443.722	2	885.429	IADYNYK	87	93	0	885.423	0.006	6.709	Trypsin
12681T004	41.37	1133.528	2	2265.042	LPDDFTGCVIAWNSNNLDSK	94	113	0	2265.037	0.005	2.071	Trypsin
12681T004	27.34	609.802	2	1217.589	VGGNYNYLYR	114	123	0	1217.583	0.006	4.906	Trypsin
12681T004	13.20	1367.717	2	2733.420	VGGNYNYLYRLFRKSNLKPFER	114	135	3	2733.450	-0.030	-11.010	Trypsin
12681T004	11.81	282.188	2	562.362	LFRK	124	127	1	562.359	0.003	5.864	Trypsin
12681T004	16.08	218.140	2	434.266	LFR	124	126	0	434.264	0.002	3.640	Trypsin
12681T004	16.05	559.821	2	1117.627	KSNLKPFER	127	135	1	1117.624	0.003	2.633	Trypsin
12681T004	19.55	495.774	2	989.534	SNLKPFER	128	135	0	989.529	0.004	4.258	Trypsin
12681T004	42.54	1019.068	2	2036.121	VVVLSFELLHAPATVCGPK	179	197	0	2036.113	0.008	4.003	Trypsin
12681T004	40.14	1083.112	2	2164.210	VVVLSFELLHAPATVCGPKK	179	198	1	2164.208	0.002	0.902	Trypsin
12681T004	27.85	1288.586	2	2575.158	STNGGDGGDGGDGGAHIVMVDAYKPTK	199	225	0	2575.161	-0.003	-1.203	Trypsin
12681T004	36.11	525.751	2	1049.487	ITNLCPFGE	1	9	0	1049.485	0.002	1.591	Glu-C
12681T004	22.72	698.318	2	1394.622	IYQAGSTPCNGVE	141	153	0	1394.614	0.008	5.750	Glu-C
12681T005	32.43	557.281	2	1112.548	FASVYAWNR	16	24	0	1112.540	0.007	6.601	Trypsin
12681T005	28.01	621.325	2	1240.635	FASVYAWNRK	16	25	1	1240.635	-0.001	-0.551	Trypsin
12681T005	18.59	1329.137	2	2656.259	KRISNCVADYSVLYNSASFSTFK	25	47	2	2656.296	-0.036	-13.574	Trypsin
12681T005	41.82	1265.110	2	2528.206	RISNCVADYSVLYNSASFSTFK	26	47	1	2528.201	0.005	2.013	Trypsin
12681T005	45.36	1187.057	2	2372.100	ISNCVADYSVLYNSASFSTFK	27	47	0	2372.099	0.000	0.176	Trypsin
12681T005	43.62	1633.260	2	3264.506	ISNCVADYSVLYNSASFSTFKCYGVSPTK	27	55	1	3264.511	-0.005	-1.552	Trypsin

12681T005	16.75	456.216	2	910.418	CYGVSPTK	48	55	0	910.422	-0.004	-4.501	Trypsin
12681T005	49.90	1024.004	2	2045.993	LNDLCFTNVYADSFVIR	56	72	0	2045.988	0.005	2.268	Trypsin
12681T005	45.24	1302.132	2	2602.249	LNDLCFTNVYADSFVIRGDEVR	56	77	1	2602.249	0.000	0.061	Trypsin
12681T005	18.12	728.382	2	1454.749	GDEVRQIAPGQTGK	73	86	1	1454.748	0.001	0.890	Trypsin
12681T005	12.22	450.253	2	898.491	QIAPGQTGK	78	86	0	898.487	0.004	4.377	Trypsin
12681T005	51.43	883.946	2	1765.878	QIAPGQTGKIADYNYK	78	93	1	1765.900	-0.022	-12.236	Trypsin
12681T005	44.71	1567.235	2	3132.455	IADYNYKLPDDFTGCVIAWNSNNLDSK	87	113	1	3132.450	0.005	1.603	Trypsin
12681T005	18.69	443.721	2	885.428	IADYNYK	87	93	0	885.423	0.005	5.456	Trypsin
12681T005	43.27	1133.530	2	2265.046	LPDDFTGCVIAWNSNNLDSK	94	113	0	2265.037	0.009	3.889	Trypsin
12681T005	28.03	609.802	2	1217.590	VGGNYNYLYR	114	123	0	1217.583	0.007	5.757	Trypsin
12681T005	9.80	282.189	2	562.362	LFRK	124	127	1	562.359	0.003	5.923	Trypsin
12681T005	15.59	218.141	2	434.267	LFR	124	126	0	434.264	0.003	5.941	Trypsin
12681T005	16.22	559.823	2	1117.632	KSNLKPFER	127	135	1	1117.624	0.008	7.016	Trypsin
12681T005	19.60	495.776	2	989.538	SNLKPFER	128	135	0	989.529	0.009	8.966	Trypsin
12681T005	44.45	1019.071	2	2036.128	VVVLSFELLHAPATVCGPK	179	197	0	2036.113	0.015	7.262	Trypsin
12681T005	42.06	1083.113	2	2164.211	VVVLSFELLHAPATVCGPKK	179	198	1	2164.208	0.003	1.515	Trypsin
12681T005	28.83	1288.590	2	2575.165	STNGGDGGDGGDGGAHIVMVDAYKPTK	199	225	0	2575.161	0.004	1.663	Trypsin
12681T005	35.46	525.749	2	1049.483	ITNLCPFGE	1	9	0	1049.485	-0.002	-1.931	Glu-C
12681T005	22.98	698.316	2	1394.618	IYQAGSTPCNGVE	141	153	0	1394.614	0.004	2.788	Glu-C

Supplementary Table S5: Identified Glycans from Sample Batch No. 12681T002

Glycoform Mass	Glycan Structure	Glycan Type	Peptide Mass [M]	Peptide Sequence	Theoretical Glycopeptide Mass	Δmass (Dalton)
527.185	$(Hex)_2$ $(HexNAc)_1$	-	1737.851	ITNLCPFGEVFNATR	2266.043	0
656.228	$(Hex)_1$ $(HexNAc)_1$ $(NeuAc)_1$	-	1680.829	ITNLCPFGEVFNATR	2338.065	-0.001
846.312	$(Hex)_1$ $(HexNAc)_2$ $(Deoxyhexose)_1$ $(Pent)_1$	-	1680.829	ITNLCPFGEVFNATR	2528.149	0
851.291	$(Hex)_4$ $(HexNAc)_1$	-	1737.851	ITNLCPFGEVFNATR	2590.149	0.002
1008.365	$(Hex)_2$ $(HexNAc)_2$ $(Deoxyhexose)_1$ $(Pent)_1$	-	1737.851	ITNLCPFGEVFNATR	2747.223	0
1170.417	$(Hex)_3$ $(HexNAc)_2$ $(Deoxyhexose)_1$ $(Pent)_1$	-	1680.829	ITNLCPFGEVFNATR	2852.254	0
1175.396	$(Hex)_6$ $(HexNAc)_1$	-	1737.851	ITNLCPFGEVFNATR	2914.254	0.002
1332.47	$(Hex)_4$ $(HexNAc)_2$ $(Deoxyhexose)_1$ $(Pent)_1$	-	1680.829	ITNLCPFGEVFNATR	3014.307	0
1346.486	$(Hex)_4$ $(HexNAc)_2$ $(Deoxyhexose)_2$	-	1737.851	ITNLCPFGEVFNATR	3085.344	0
1387.512	$(HexNAc)_1$ $(Deoxyhexose)_2$ + $(Man)_3(GlcNAc)_2$	hybrid/complex	1680.829	ITNLCPFGEVFNATR	3069.349	0.005
1403.507	$(Hex)_1$ $(HexNAc)_1$ $(Deoxyhexose)_1$ + $(Man)_3(GlcNAc)_2$	hybrid/complex	1680.829	ITNLCPFGEVFNATR	3085.344	0.001
1403.507	$(Hex)_1$ $(HexNAc)_1$ $(Deoxyhexose)_1$ + $(Man)_3(GlcNAc)_2$	hybrid/complex	1737.851	ITNLCPFGEVFNATR	3142.365	0.005
1460.529	$(Hex)_1$ $(HexNAc)_2$ + $(Man)_3(GlcNAc)_2$	hybrid/complex	1680.829	ITNLCPFGEVFNATR	3142.366	0.004
2076.75	$(Hex)_3$ $(HexNAc)_2$ $(Deoxyhexose)_2$ + $(Man)_3(GlcNAc)_2$	hybrid/complex	1680.829	ITNLCPFGEVFNATR	3758.587	0.005

Supplementary Table S6: Identified Glycans from Sample Batch No. 12681T003

Glycoform Mass	Glycan Structure	Glycan Type	Peptide Mass [M]	Peptide Sequence	Theoretical Glycopeptide Mass	Δmass (Dalton)
527.185	$(Hex)_2$ $(HexNAc)_1$	-	1737.851	ITNLCPFGEVFNATR	2266.043	0
656.228	$(Hex)_1$ $(HexNAc)_1$ $(NeuAc)_1$	-	1680.829	ITNLCPFGEVFNATR	2338.065	-0.001
730.264	$(Hex)_2$ $(HexNAc)_2$	-	1737.851	ITNLCPFGEVFNATR	2469.122	0.003
846.312	$(Hex)_1$ $(HexNAc)_2$ $(Deoxyhexose)_1$ $(Pent)_1$	-	1737.851	ITNLCPFGEVFNATR	2585.17	0.001
972.284	$(Hex)_3$ $(HexNAc)_2$ $(Phos)_1$	-	1737.851	ITNLCPFGEVFNATR	2711.142	0.004
1008.365	$(Hex)_2$ $(HexNAc)_2$ $(Deoxyhexose)_1$ $(Pent)_1$	-	1680.829	ITNLCPFGEVFNATR	2690.202	0.001
1022.38	$(Hex)_2$ $(HexNAc)_2$ $(Deoxyhexose)_2$	-	1680.829	ITNLCPFGEVFNATR	2704.217	0.003
1170.417	$(Hex)_3$ $(HexNAc)_2$ $(Deoxyhexose)_1$ $(Pent)_1$	-	1680.829	ITNLCPFGEVFNATR	2852.254	0.002
1175.396	$(Hex)_6$ $(HexNAc)_1$	-	1737.851	ITNLCPFGEVFNATR	2914.254	0.003
1186.412	$(Hex)_4$ $(HexNAc)_2$ $(Pent)_1$	-	1737.851	ITNLCPFGEVFNATR	2925.27	0
1332.47	$(Hex)_4$ $(HexNAc)_2$ $(Deoxyhexose)_1$ $(Pent)_1$	-	1680.829	ITNLCPFGEVFNATR	3014.307	0.003
1346.486	$(Hex)_4$ $(HexNAc)_2$ $(Deoxyhexose)_2$	-	1737.851	ITNLCPFGEVFNATR	3085.344	0.004
1387.512	$(HexNAc)_1$ $(Deoxyhexose)_2$ +	hybrid/complex	1737.851	ITNLCPFGEVFNATR	3126.37	0.003
1403.507	$(Hex)_1$ $(HexNAc)_1$ $(Deoxyhexose)_1$ +	hybrid/complex	1680.829	ITNLCPFGEVFNATR	3085.344	0.005
1444.534	$(HexNAc)_2$ $(Deoxyhexose)_1$ +	hybrid/complex	1680.829	ITNLCPFGEVFNATR	3126.371	0.002
1581.555	$(Hex)_3$ $(HexNAc)_1$ + $(Man)_3(GlcNAc)_2$	hybrid/complex	1737.851	ITNLCPFGEVFNATR	3320.413	0
1752.645	$(Hex)_1$ $(HexNAc)_2$ $(Deoxyhexose)_2$ +	hybrid/complex	1737.851	ITNLCPFGEVFNATR	3491.503	-0.002
1793.671	$(HexNAc)_3$ $(Deoxyhexose)_2$ +	hybrid/complex	1737.851	ITNLCPFGEVFNATR	3532.529	0
1809.666	$(Hex)_1$ $(HexNAc)_3$ $(Deoxyhexose)_1$ +	hybrid/complex	1680.829	ITNLCPFGEVFNATR	3491.503	-0.002
1850.693	$(HexNAc)_4$ $(Deoxyhexose)_1$ +	hybrid/complex	1680.829	ITNLCPFGEVFNATR	3532.53	0
2028.74	$(Hex)_2$ $(HexNAc)_4$ + $(Man)_3(GlcNAc)_2$	hybrid/complex	1737.851	ITNLCPFGEVFNATR	3767.598	0.005
2076.75	$(Hex)_3$ $(HexNAc)_2$ $(Deoxyhexose)_2$ +	hybrid/complex	1680.829	ITNLCPFGEVFNATR	3758.587	0.003
2204.772	$(Hex)_2$ $(HexNAc)_2$ $(NeuAc)_2$ +	hybrid/complex	1737.851	ITNLCPFGEVFNATR	3943.63	-0.002
2205.793	$(Hex)_2$ $(HexNAc)_2$ $(Deoxyhexose)_2$	hybrid/complex	1737.851	ITNLCPFGEVFNATR	3944.651	-0.003
2238.803	$(Hex)_4$ $(HexNAc)_2$ $(Deoxyhexose)_2$ +	hybrid/complex	1680.829	ITNLCPFGEVFNATR	3920.64	0.002
2262.814	$(Hex)_2$ $(HexNAc)_3$ $(Deoxyhexose)_1$	hybrid/complex	1680.829	ITNLCPFGEVFNATR	3944.651	-0.002
2465.894	$(Hex)_2$ $(HexNAc)_4$ $(Deoxyhexose)_1$	hybrid/complex	1737.851	ITNLCPFGEVFNATR	4204.752	-0.003
2522.915	$(Hex)_2$ $(HexNAc)_5$ $(NeuAc)_1$ +	hybrid/complex	1680.829	ITNLCPFGEVFNATR	4204.751	-0.002
2586.92	$(Hex)_4$ $(HexNAc)_3$ $(Deoxyhexose)_1$	hybrid/complex	1680.829	ITNLCPFGEVFNATR	4268.757	0.001
3181.184	$(Hex)_3$ $(HexNAc)_6$ $(Deoxyhexose)_4$ +	hybrid/complex	1680.829	ITNLCPFGEVFNATR	4863.021	0

Supplementary Table S7: Identified Glycans from Sample Batch No. 12681T004

Glycoform Mass	Glycan Structure	Glycan Type	Peptide Mass [M]	Peptide Sequence	Theoretical Glycopeptide Mass	Δmass (Dalton)
527.185	$(Hex)_2$ $(HexNAc)_1$	-	1737.851	ITNLCPFGEVFNATR	2266.043	0
656.228	$(Hex)_1$ $(HexNAc)_1$ $(NeuAc)_1$	-	1737.851	ITNLCPFGEVFNATR	2395.086	-0.003
846.312	$(Hex)_1$ $(HexNAc)_2$ $(Deoxyhexose)_1$ $(Pent)_1$	-	1737.851	ITNLCPFGEVFNATR	2585.17	0
1008.365	$(Hex)_2$ $(HexNAc)_2$ $(Deoxyhexose)_1$ $(Pent)_1$	-	1680.829	ITNLCPFGEVFNATR	2690.202	-0.001
1013.343	$(Hex)_5$ $(HexNAc)_1$	-	1680.829	ITNLCPFGEVFNATR	2695.18	0
1022.38	$(Hex)_2$ $(HexNAc)_2$ $(Deoxyhexose)_2$	-	1680.829	ITNLCPFGEVFNATR	2704.217	0.002
1170.417	$(Hex)_3$ $(HexNAc)_2$ $(Deoxyhexose)_1$ $(Pent)_1$	-	1680.829	ITNLCPFGEVFNATR	2852.254	0.005
1216.423	$(Hex)_2$ + $(Man)_3(GlcNAc)_2$	high_man	1737.851	ITNLCPFGEVFNATR	2955.281	-0.002
1332.47	$(Hex)_4$ $(HexNAc)_2$ $(Deoxyhexose)_1$ $(Pent)_1$	-	1680.829	ITNLCPFGEVFNATR	3014.307	-0.003
1378.476	$(Hex)_3$ + $(Man)_3(GlcNAc)_2$	high_man	1737.851	ITNLCPFGEVFNATR	3117.334	0.003
1387.512	$(HexNAc)_1$ $(Deoxyhexose)_2$ + $(Man)_3(GlcNAc)_2$	hybrid/complex	1680.829	ITNLCPFGEVFNATR	3069.349	0.003
1540.528	$(Hex)_4$ + $(Man)_3(GlcNAc)_2$	high_man	1737.851	ITNLCPFGEVFNATR	3279.386	0.003
1651.597	$(HexNAc)_1$ $(Deoxyhexose)_2$ $(Pent)_2$ + $(Man)_3(GlcNAc)_2$	hybrid/complex	1680.829	ITNLCPFGEVFNATR	3333.434	-0.004
1898.703	$(Hex)_1$ $(HexNAc)_2$ $(Deoxyhexose)_3$ + $(Man)_3(GlcNAc)_2$	hybrid/complex	1737.851	ITNLCPFGEVFNATR	3637.561	0.001
1955.724	$(Hex)_1$ $(HexNAc)_3$ $(Deoxyhexose)_2$ + $(Man)_3(GlcNAc)_2$	hybrid/complex	1680.829	ITNLCPFGEVFNATR	3637.561	0.002
1971.719	$(Hex)_2$ $(HexNAc)_3$ $(Deoxyhexose)_1$ + $(Man)_3(GlcNAc)_2$	hybrid/complex	1737.851	ITNLCPFGEVFNATR	3710.577	0
2026.687	$(Hex)_7$ + $(Man)_3(GlcNAc)_2$	high_man	1737.851	ITNLCPFGEVFNATR	3765.545	-0.004
2028.74	$(Hex)_2$ $(HexNAc)_4$ + $(Man)_3(GlcNAc)_2$	hybrid/complex	1680.829	ITNLCPFGEVFNATR	3710.577	0
2035.681	$(Hex)_1$ $(HexNAc)_3$ $(Deoxyhexose)_2$ $(Sulph)_1$ + $(Man)_3(GlcNAc)_2$	hybrid/complex	1737.851	ITNLCPFGEVFNATR	3774.539	0
2076.75	$(Hex)_3$ $(HexNAc)_2$ $(Deoxyhexose)_2$ + $(Man)_3(GlcNAc)_2$	hybrid/complex	1680.829	ITNLCPFGEVFNATR	3758.587	0
2188.74	$(Hex)_8$ + $(Man)_3(GlcNAc)_2$	high_man	1737.851	ITNLCPFGEVFNATR	3927.598	-0.003
2238.803	$(Hex)_4$ $(HexNAc)_2$ $(Deoxyhexose)_2$ + $(Man)_3(GlcNAc)_2$	hybrid/complex	1680.829	ITNLCPFGEVFNATR	3920.64	0.005
2238.803	$(Hex)_4$ $(HexNAc)_2$ $(Deoxyhexose)_2$ + $(Man)_3(GlcNAc)_2$	hybrid/complex	1737.851	ITNLCPFGEVFNATR	3977.661	0
2295.825	$(Hex)_4$ $(HexNAc)_3$ $(Deoxyhexose)_1$ + $(Man)_3(GlcNAc)_2$	hybrid/complex	1680.829	ITNLCPFGEVFNATR	3977.662	-0.001

Supplementary Table S8: Identified Glycans from Sample Batch No. 12681T005

Glycoform Mass	Glycan Structure	Glycan Type	Peptide Mass [M]	Peptide Sequence	Theoretical Glycopeptide Mass	Δmass (Dalton)
527.185	$(Hex)_2$ $(HexNAc)_1$	-	1737.851	ITNLCPFGEVFNATR	2266.043	-0.001
656.228	$(Hex)_1$ $(HexNAc)_1$ $(NeuAc)_1$	-	1680.829	ITNLCPFGEVFNATR	2338.065	0
846.312	$(Hex)_1$ $(HexNAc)_2$ $(Deoxyhexose)_1$ $(Pent)_1$	-	1680.829	ITNLCPFGEVFNATR	2528.149	0
876.322	$(Hex)_2$ $(HexNAc)_2$ $(Deoxyhexose)_1$	-	1737.851	ITNLCPFGEVFNATR	2615.18	0.002
892.317	$(Hex)_3$ $(HexNAc)_2$	-	1680.829	ITNLCPFGEVFNATR	2574.154	0
933.344	$(Hex)_2$ $(HexNAc)_3$	-	1680.829	ITNLCPFGEVFNATR	2615.181	0.001
1008.365	$(Hex)_2$ $(HexNAc)_2$ $(Deoxyhexose)_1$ $(Pent)_1$	-	1737.851	ITNLCPFGEVFNATR	2747.223	0.003
1170.417	$(Hex)_3$ $(HexNAc)_2$ $(Deoxyhexose)_1$ $(Pent)_1$	-	1680.829	ITNLCPFGEVFNATR	2852.254	0.004
1332.47	$(Hex)_4$ $(HexNAc)_2$ $(Deoxyhexose)_1$ $(Pent)_1$	-	1680.829	ITNLCPFGEVFNATR	3014.307	-0.001
1548.545	$(Hex)_1$ $(HexNAc)_1$ $(NeuAc)_1$ + $(Man)_3(GlcNAc)_2$	hybrid/complex	1680.829	ITNLCPFGEVFNATR	3230.382	-0.001
1622.582	$(Hex)_2$ $(HexNAc)_2$ + $(Man)_3(GlcNAc)_2$	hybrid/complex	1737.851	ITNLCPFGEVFNATR	3361.44	-0.002
1661.555	$(Hex)_9$ $(HexNAc)_1$	-	1680.829	ITNLCPFGEVFNATR	3343.392	-0.002
1854.676	$(HexNAc)_2$ $(Deoxyhexose)_2$ $(Pent)_2$ + $(Man)_3(GlcNAc)_2$	hybrid/complex	1680.829	ITNLCPFGEVFNATR	3536.513	-0.002
2026.687	$(Hex)_7$ + $(Man)_3(GlcNAc)_2$	high_man	1737.851	ITNLCPFGEVFNATR	3765.545	-0.002
2110.794	$(HexNAc)_6$ + $(Man)_3(GlcNAc)_2$	hybrid/complex	1737.851	ITNLCPFGEVFNATR	3849.652	-0.003
2142.809	$(HexNAc)_4$ $(Deoxyhexose)_3$ + $(Man)_3(GlcNAc)_2$	hybrid/complex	1737.851	ITNLCPFGEVFNATR	3881.667	0.002
2188.74	$(Hex)_8$ + $(Man)_3(GlcNAc)_2$	high_man	1737.851	ITNLCPFGEVFNATR	3927.598	0.004
2262.814	$(Hex)_2$ $(HexNAc)_3$ $(Deoxyhexose)_1$ $(NeuAc)_1$ + $(Man)_3(GlcNAc)_2$	hybrid/complex	1737.851	ITNLCPFGEVFNATR	4001.672	0
2319.836	$(Hex)_2$ $(HexNAc)_4$ $(NeuAc)_1$ + $(Man)_3(GlcNAc)_2$	hybrid/complex	1680.829	ITNLCPFGEVFNATR	4001.673	0
2459.931	$(HexNAc)_7$ $(Deoxyhexose)_1$ + $(Man)_3(GlcNAc)_2$	hybrid/complex	1737.851	ITNLCPFGEVFNATR	4198.789	0
2516.952	$(HexNAc)_8$ + $(Man)_3(GlcNAc)_2$	hybrid/complex	1680.829	ITNLCPFGEVFNATR	4198.789	0

Supplementary Table S9: Batch release summary

Test Name	Specification/ Acceptance Criteria	Unit	Batch No.			
			12681T002	12681T003	12681T004	12681T005
Appearance	Clear Colourless to yellowish solution free from visible particles	NA	Clear Colourless solution free from visible particles	Clear Colourless solution free from visible particles	Clear Colourless solution free from visible particles	Clear Colourless solution free from visible particles
pH	7.00 -8.00	NA	7.17	7.26	7.2	7.22
Identity By ELISA	Positive Reaction for RBD Spytag with anti-RBD Antibody In ELISA	NA	Positive reaction for RBD Spytag with anti-RBD Antibody In ELISA	Positive reaction for RBD Spytag with anti-RBD Antibody In ELISA	Positive reaction for RBD Spytag with anti-RBD Antibody In ELISA	Positive reaction for RBD Spytag with anti-RBD Antibody In ELISA
Purity By SDS -PAGE	Not less than 90%	%	99.81	100	100	100
Purity By HPLC	Not Less than 90 %	%	100	100	100	100
Identity By SDS PAGE and Western Blotting	Molecular weight of main band should not be less than 24 kDa and band should be identified by western Blot	NA	Molecular weight of main band is 32.18 kDa and band is identified	Molecular weight of main band is 27.07 kDa and band is identified	Molecular weight of main band is 27.31 kDa and band is identified	Molecular weight of main is 27.67 kDa and band is identified
Protein Content	Not less than 10 mg/mL	mg/mL	13.48	16.78	15.01	13.29
Endotoxin Content	Less than or equal to 4 EU/MCG Protein	EU/µG Protein	<0.05	<0.05	<0.05	<0.05
Antigenic Ratio	Not less than 0.700	NA	0.928	0.973	1.016	1.341
Antigenic Content	Not less than 7 mg/mL	mg/mL	12.51	16.33	15.25	17.82
Bioburden	Less than or equal to 10CFU/10ml	CFU/10mL	0	0	0	0
Host Cell Protein (HCP) content	Less than or equal to 5% of Protein	%	0.01	0.01	0.01	0.01
Host cell DNA (HCD) Content	Less than or equal to 1Ng/MCG of Protein	ng/ µg	0.1	0.08	0.09	0.07

Supplementary Figure S1. Mixed-mode chromatography (B. No. 12680T002)

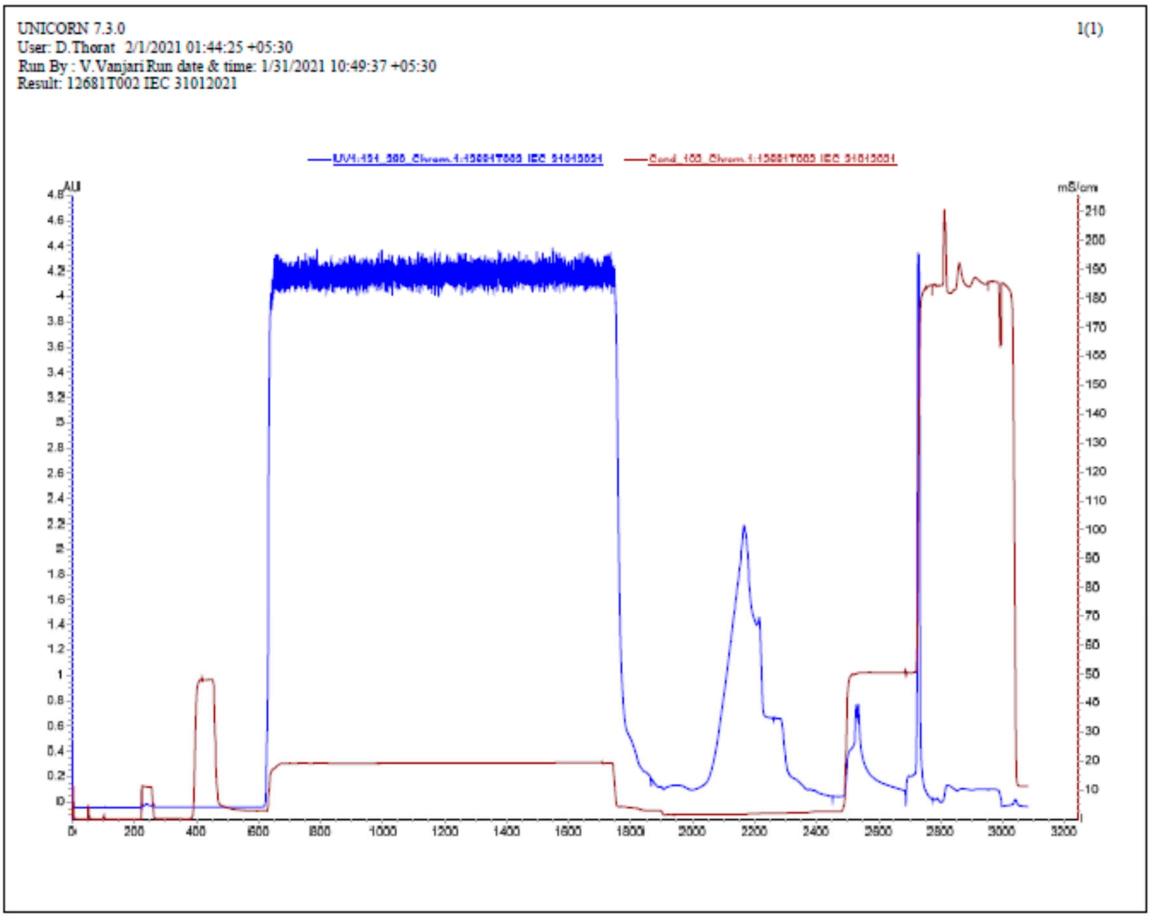

Supplementary Figure S2. Mixed-mode chromatography (B. No. 12680T003)

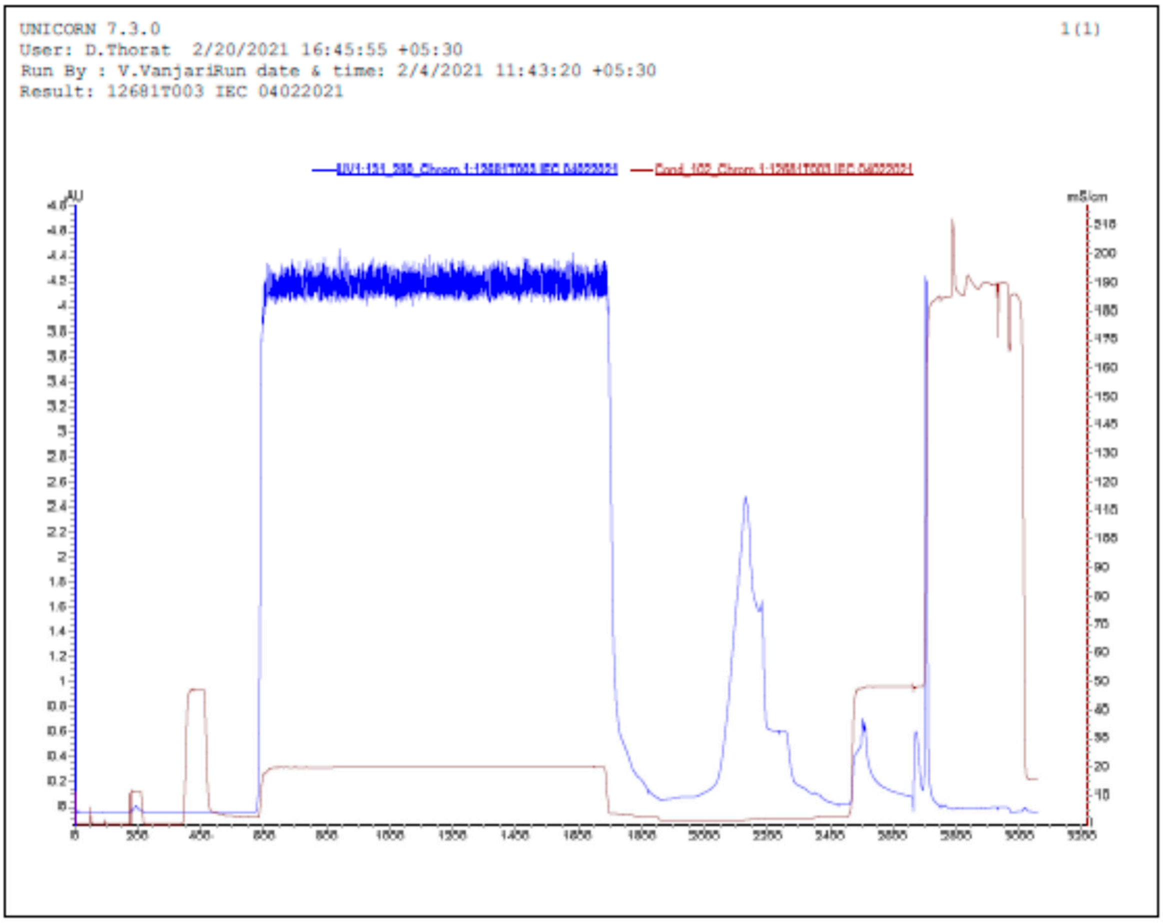

Supplementary Figure S3. Mixed-mode chromatography (B. No. 12680T004)

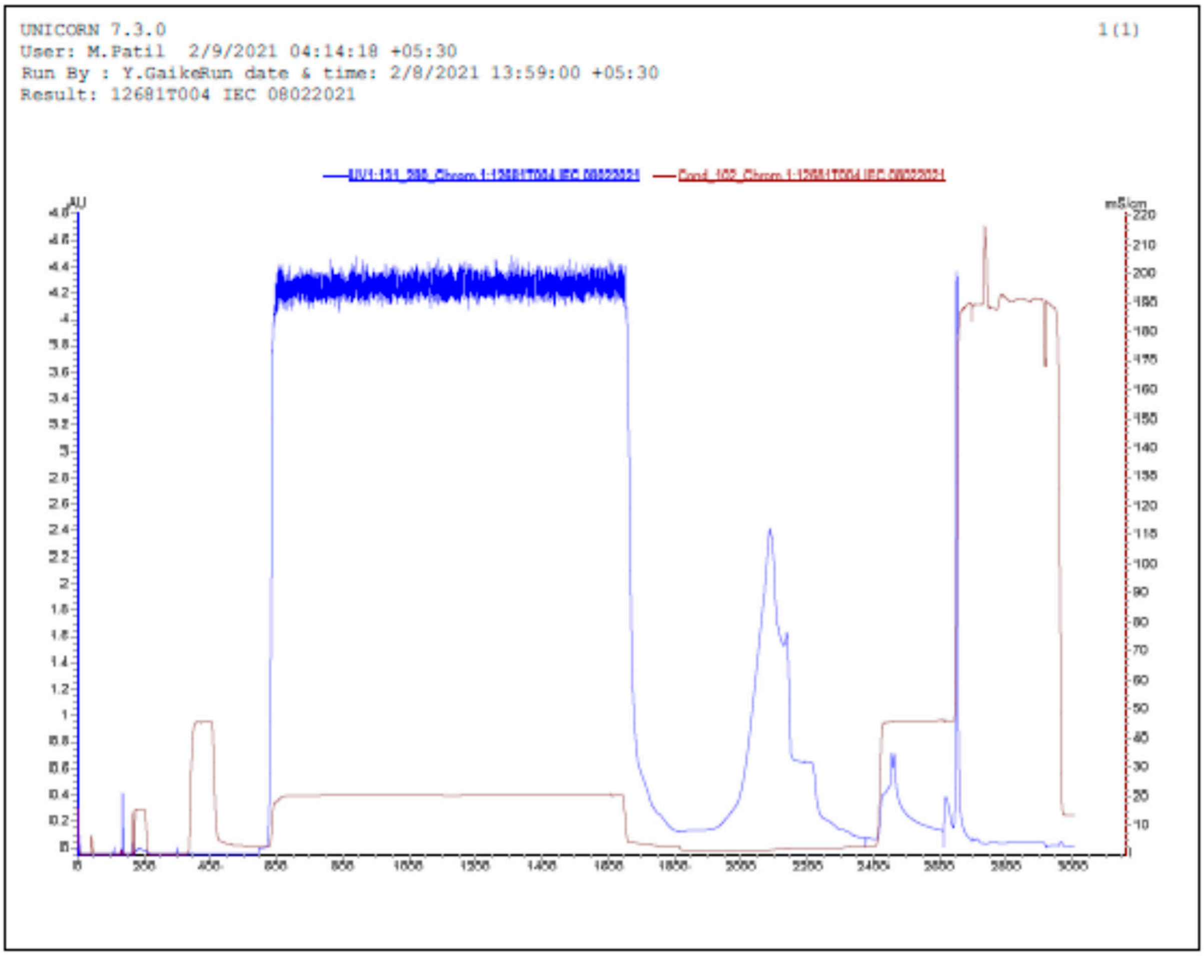

Supplementary Figure S4. Mixed-mode chromatography (B. No. 12680T005)

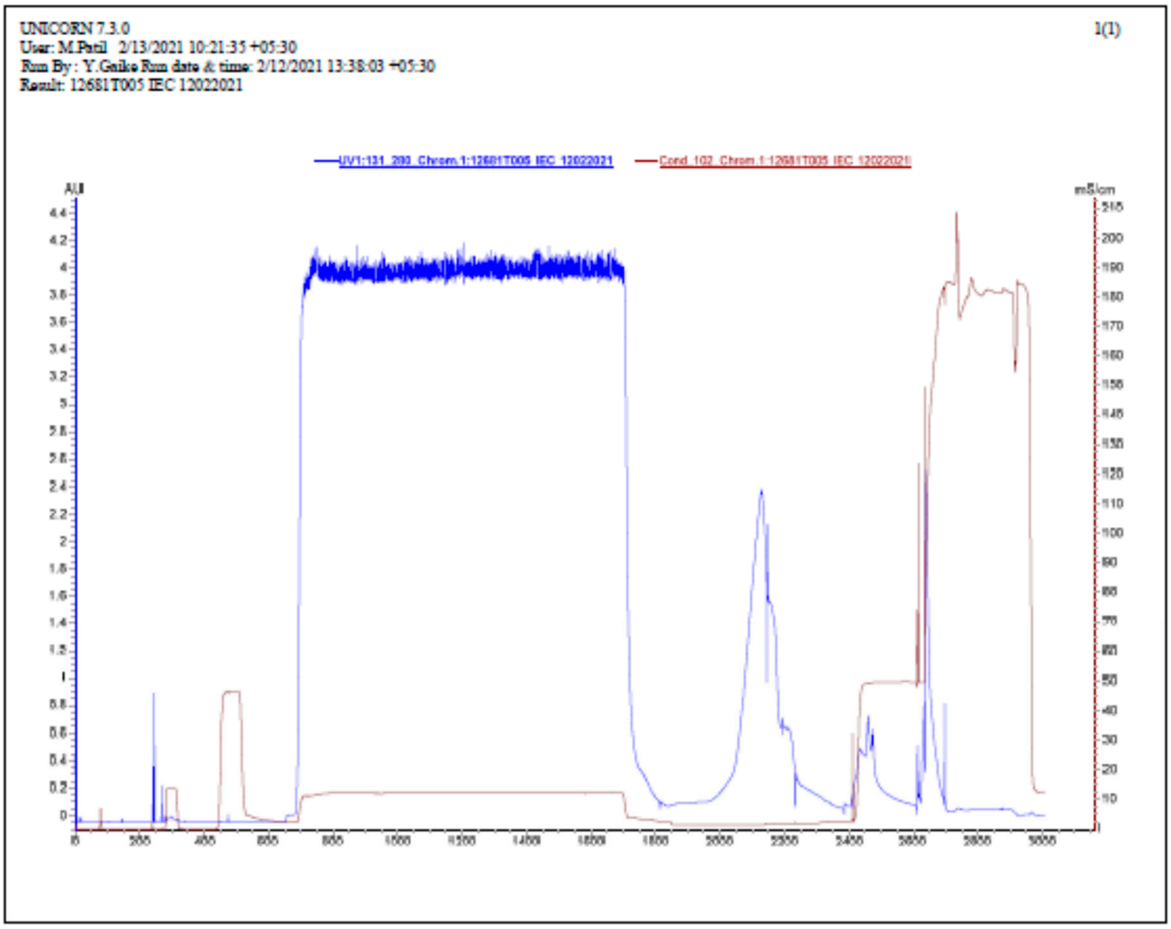

Supplementary Figure S5. Hydrophobic Interaction chromatography (B. No. 12680T002)

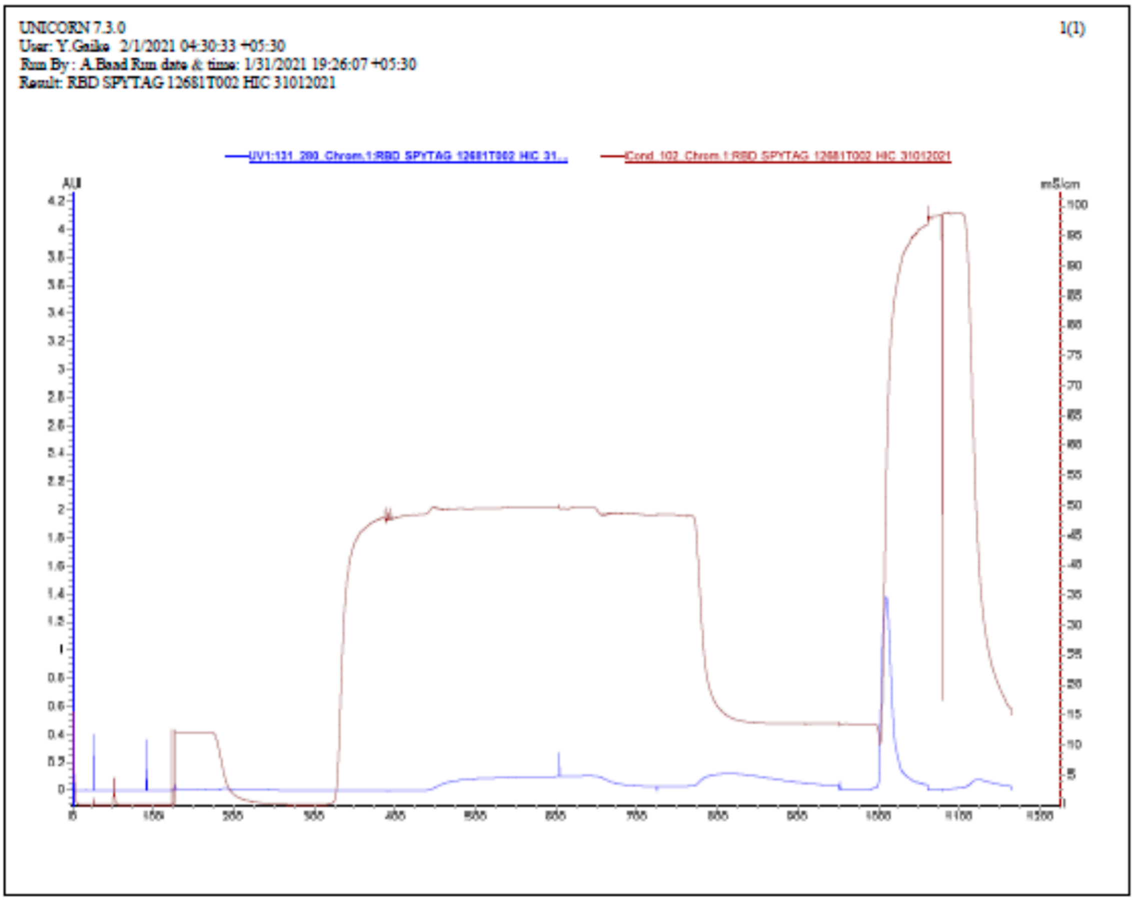

Supplementary Figure S6. Hydrophobic Interaction chromatography (B. No. 12680T003)

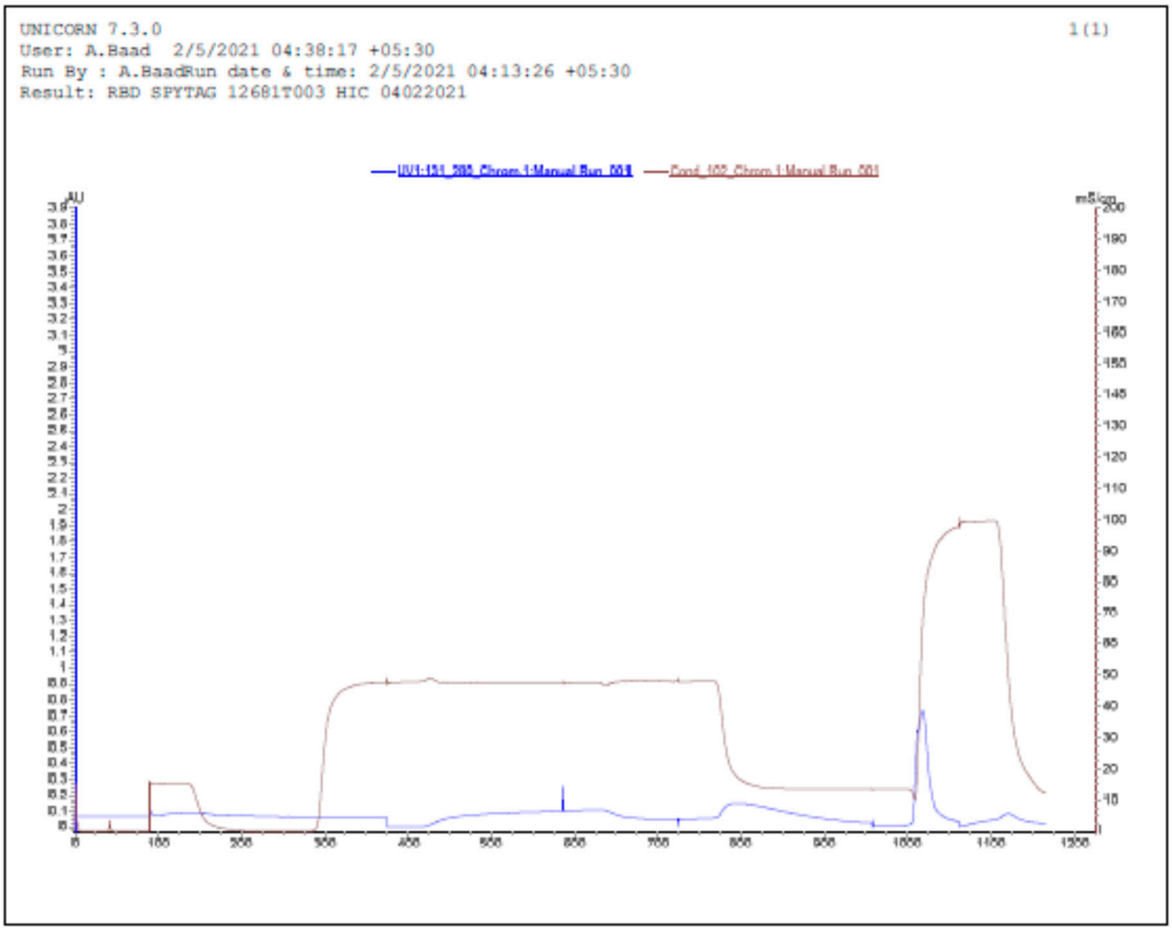

Supplementary Figure S7. Hydrophobic Interaction chromatography (B. No. 12680T004)

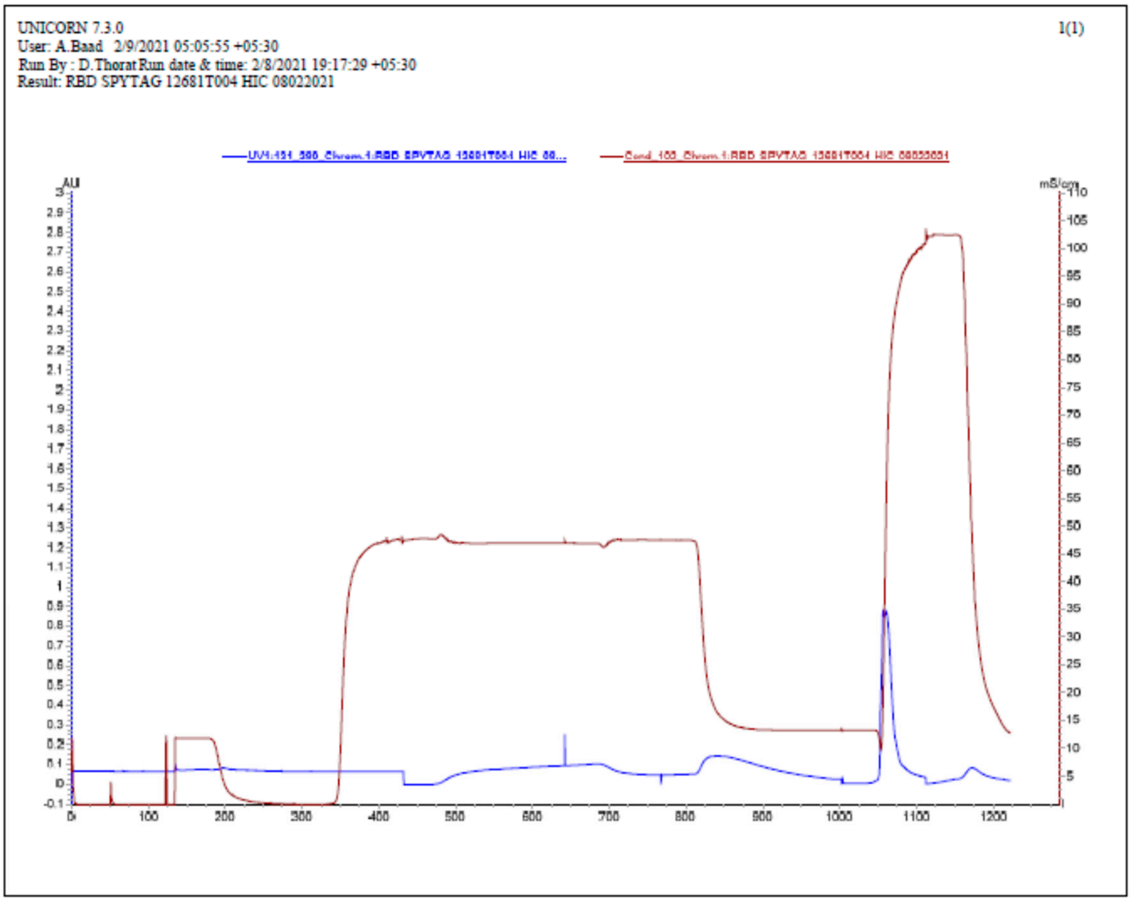

Figure S8. Hydrophobic Interaction chromatography (B. No. 12680T005)

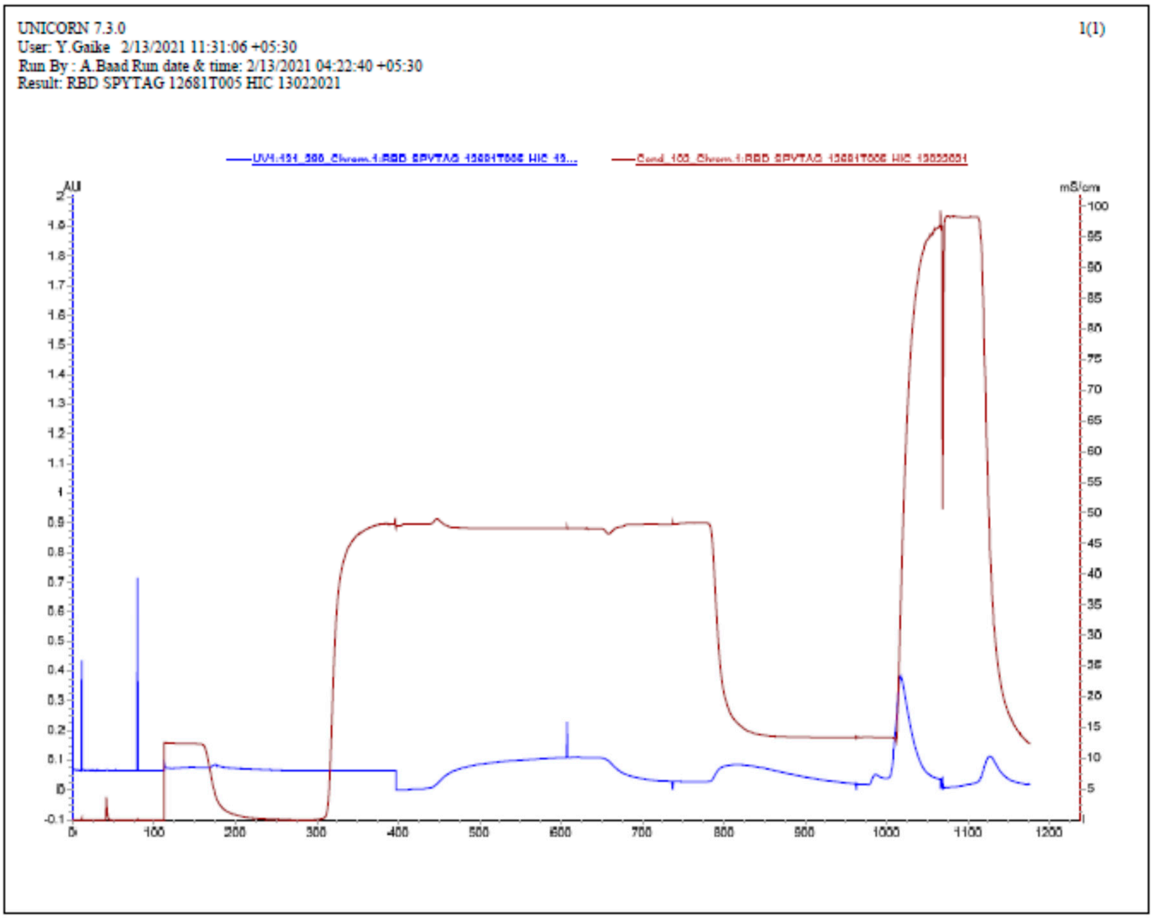

Supplementary Figure S9: Theoretical Construct of Di-Sulfide linked peptides that can be obtained upon Trypsin digestion of the protein at C terminal Arginine and lysine

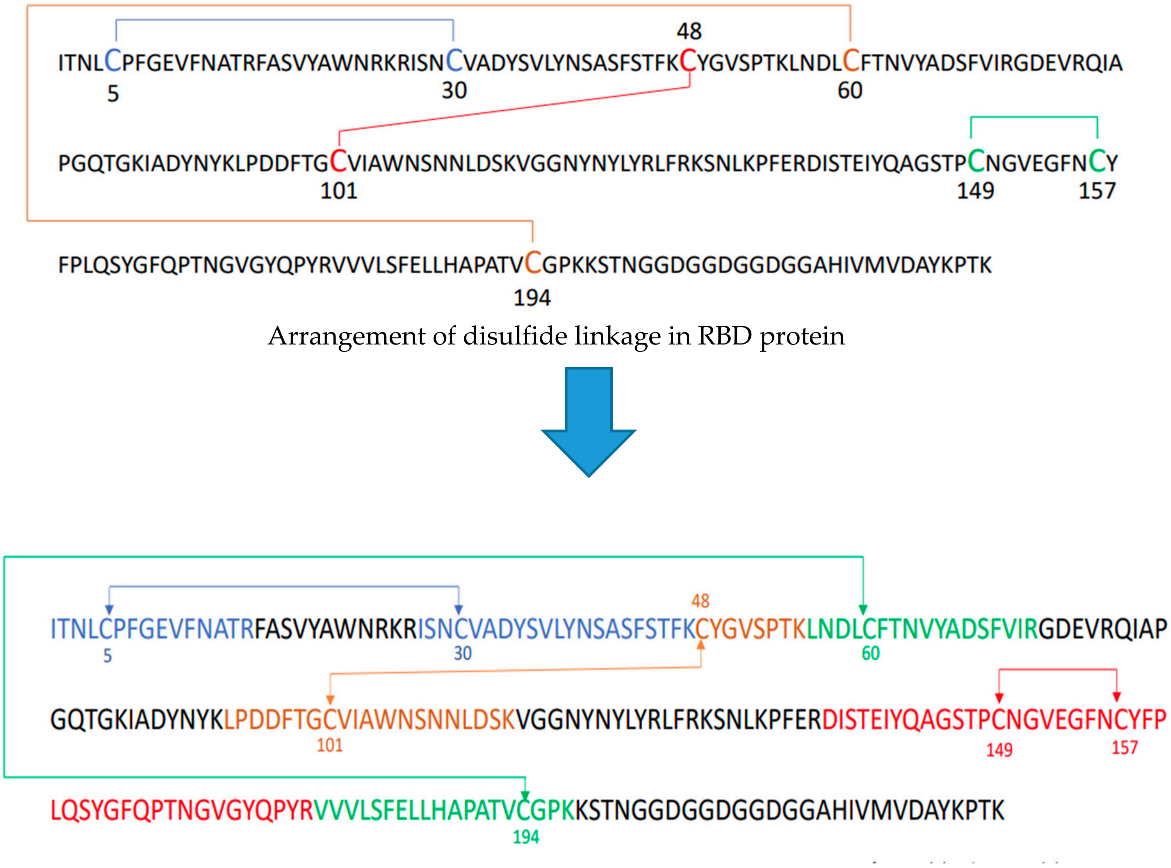

Supplementary Figure S10 m/z spectrum of Di-Sulfide linked peptides obtained from B. No. 12681T002

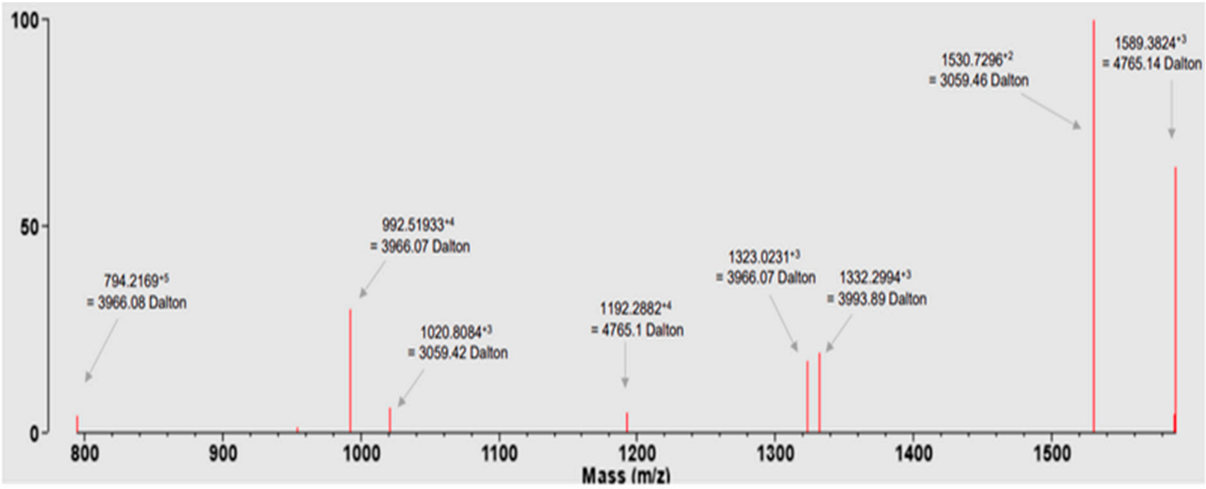

Supplementary Figure S11 m/z spectrum of Di-Sulfide linked peptides obtained from B. No. 12681T003

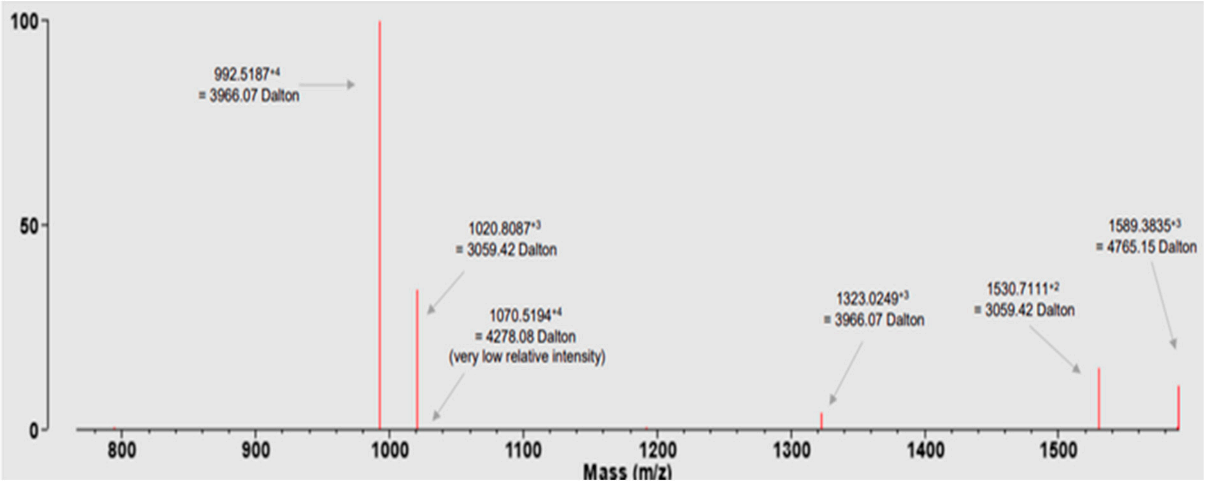

Supplementary Figure S12 m/z spectrum of Di-Sulfide linked peptides obtained from B. No. 12681T004

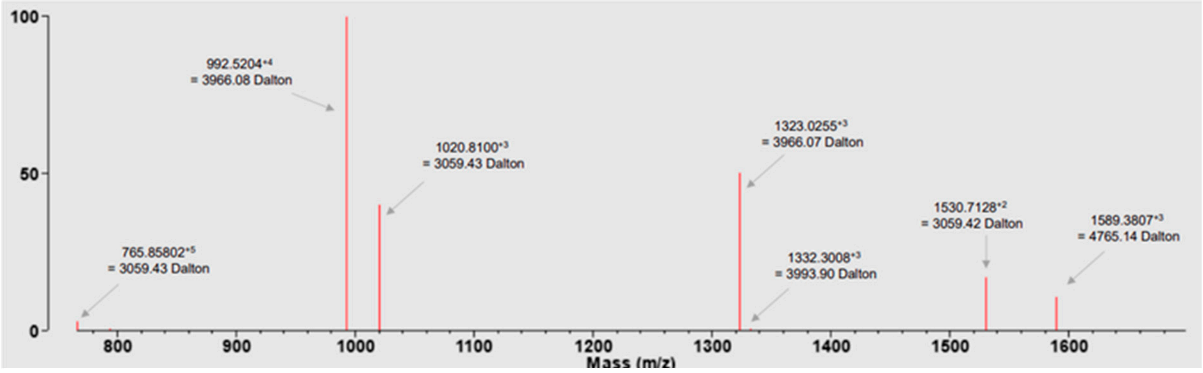

Supplementary Figure S13 m/z spectrum of Di-Sulfide linked peptides obtained from B. No. 12681T005

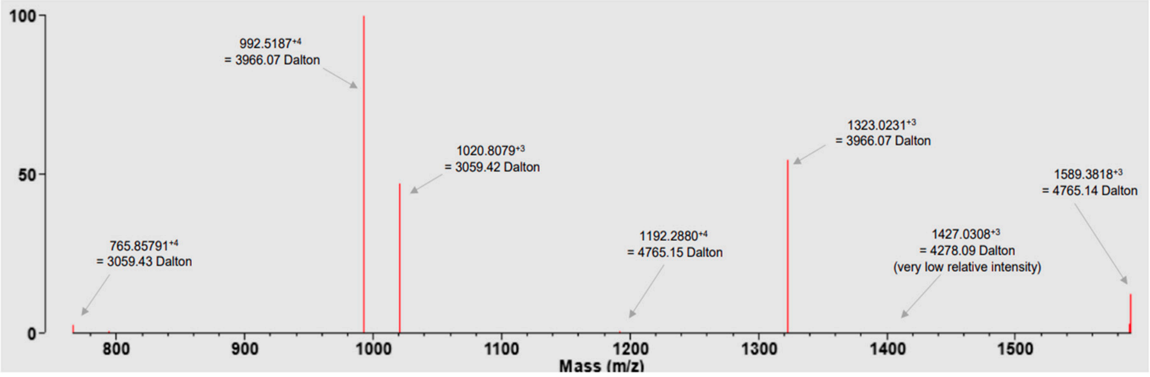

Figure S14. Deconvoluted Molecular Mass of 'RBD SARS-CoV2 Ag with Spytag' (B. No. 12680T002)

Glycosylated Reducing Conditions

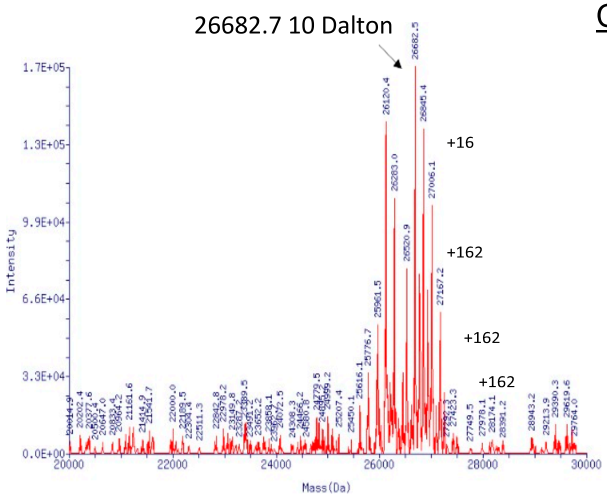

Deglycosylated Reducing Conditions

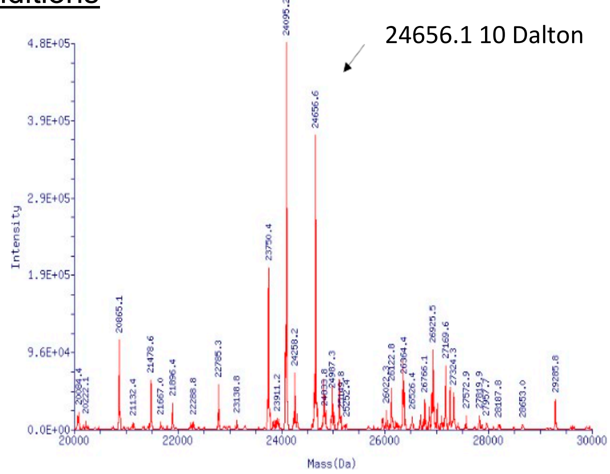

Figure S15. Deconvoluted Molecular Mass of 'RBD SARS-CoV2 Ag with Spytag' (B. No. 12680T003)

Glycosylated Reducing Conditions

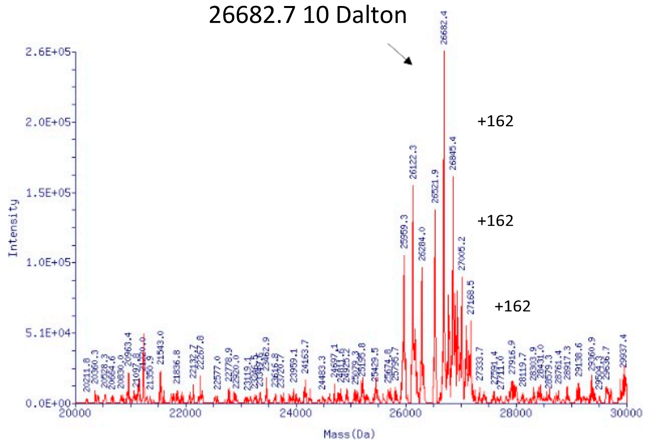

Deglycosylated Reducing

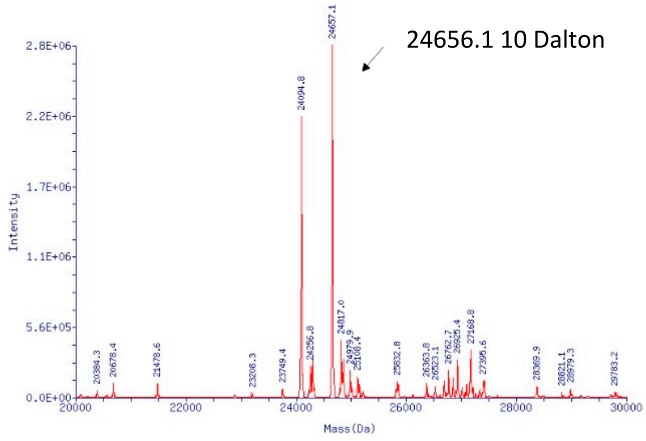

Figure S16: Sequence details of RBD construct:

(Reference: Neil, C. Dalvie.; et al. Scalable, Methanol-Free Manufacturing of the SARS-CoV-2 Receptor-Binding Domain in Engineered *Komagataella Phaffii*. Biotechnology and Bioengineering 2022, 119,2,657–62**)**

Amino Acid Sequence:

MRFPSIFTAVLFAASSALAAPVNTTTEDETAQIPAEAVIGYSDLEGDFDVAVLPFSNS
TNNGLLFINTTIASIAAKEEGVSLEKRITNLCPFGEVFNATRFASVYAWNRKRISNCV
ADYSVLYNSASFSTFKCYGVSPTKLNDLCFTNVYADSFVIRGDEVRQIAPGQTGKIA
DYNYKLPDDFTGCVIAWNSNNLDSKVGGNYNYLYRLFRKSNLKPFERDISTEIYQA
GSTPCNGVEGFNCYFPLQSYGFQPTNGVGYQPYRVVVLSFELLHAPATVCGPKKS
TGGDGGDGGDGGAHIVMVDAYKPTK

Signal Peptide	RBD protein	Linker	Spy tag

Figure S17. Expression Construct scheme and strain information:

Details: L1 to L2:pAOX1 promoter.

L2 to L3: RBD_linker_ spy tag
L3 to L4: AOX1_TT
L4 to L5: pTEF Promoter
L5 to L6:Em7 promoter, Zeocin Resistance
L6: CYC1 terminator
L-7 to L-8: pUC origin of replication
L-8 to L1:pAOX1 promoter.

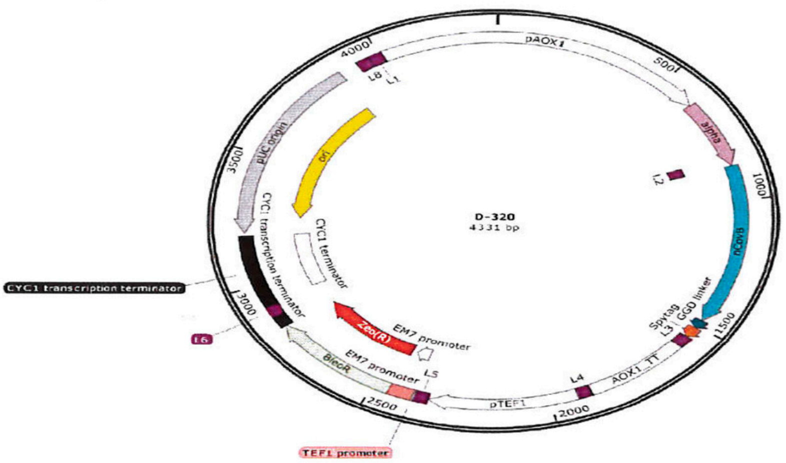

Strain Information:

Strain S-380 is derived from base strain NRRL Y-11430 with genomic modification, which has been reported before.

Strain Information RNA sequencing data is available in the NCBI Gene Expression Omnibus, accession GSE183408 (**GSE183408_phaffi_transcripts_plusTransgenes011021.fa.gz**)
